# Acquisition of high-resolution topographic information in forest environments using integrated UAV-LiDAR system: System development and field demonstration

**DOI:** 10.1016/j.heliyon.2023.e20225

**Published:** 2023-09-17

**Authors:** Shin-Kyu Choi, Ryan Angeles Ramirez, Tae-Hyuk Kwon

**Affiliations:** a Structural and Construction Technology Group, Korea Electric Power Research Institute (KEPRI), Daejeon, 34056, South Korea; b Department of Civil Engineering, University of Santo Tomas (UST), Manila, 1008, Republic of the Philippines; c Department of Civil and Environmental Engineering, Korea Advanced Institute of Science and Technology (KAIST), Daejeon, 34141, South Korea

**Keywords:** Landslide, Topographic information, UAV, LiDAR, Debris-flow barrier

## Abstract

High-resolution topographic information of landslide-prone areas plays an important role in accurate prediction and characterization of potential landslides and mitigation of landslides-associated hazards. This study presents an advanced geomorphological surveying system that integrates the light detection and ranging (LiDAR) with an unmanned aerial vehicle (UAV), a multi-rotor aerial vehicle in specific, for prediction, monitoring and forensic analysis of landslides, and for maintenance of debris-flow barriers. The test-flight over a vegetated area demonstrates that the integrated UAV-LiDAR system can provide high-resolution, three-dimensional (3D) LiDAR point clouds below canopy and vegetation in forest environments, overcoming the limitation of aerial photogrammetry and terrestrial LiDAR platforms. An algorithm is suggested to delineate the topographic information from the acquired 3D LiDAR point clouds, and the accuracy and performance of the developed UAV-LiDAR system are examined through field demonstration. Finally, two field demonstrations are presented: the forensic analysis of the recent Gokseong landslide event, and the sediment deposition monitoring for debris-flow barrier maintenance in South Korea. The developed surveying system is expected to contribute to geomorphological field surveys in vegetated, forest environments, particularly in a site that is not easily accessible.

## 1 Introduction

Landslides have occurred more frequently in recent years due to the intensified heavy rainfalls. Notable examples include landslide events that occurred in Shuicheng County, China, on July 23, 2019 [1], California, USA, on January 9, 2018 [2,3], and Mt. Woomyeon, Seoul, South Korea, on July 26–27, 2011 [4,5]. Prevention and mitigation of such catastrophic landslide-associated hazards requires understanding and predicting the landslide characteristics, such as landslide location, volume, flow velocity, flow path, runout distance, and potential damage. Reichenbach et al. [6] have conducted a cluster study on the thematic parameters for landslide prediction and it has reported that more than 70% of parameters are relevant to topographic information and they significantly impact the accuracy of landslide prediction. Accordingly, topographic information of landslide-prone areas plays a critical role in the landslide-related analyses—not only the occurrence prediction, flow analysis and vulnerability analysis, but also the countermeasure installations [[7], [8], [9], [10], [11], [12], [13], [14], [15], [16], [17]].

There are several approaches to acquire topographic information. A point-based survey method is one of the primitive methods that acquire data at selected multiple locations using hand-held equipment; however, this method is not feasible to cover large areas or inaccessible areas such as steep slopes or cliffs. Terrestrial light detection and ranging (or terrestrial LiDAR), also referred to as terrestrial laser scanning (TLS), is increasingly applied as it can obtain high-resolution three-dimensional (3D) points [[18], [19], [20], [21]]. Meanwhile, topographic points can be often lost due to any object placed in the laser propagation path of the LiDAR sensor, which is considered as limitation of the TLS method [[22], [23], [24], [25], [26]].

Thereafter, intensive efforts have been made to exploit airborne platforms (*e.g.*, airplanes, aerostatic balloons, helicopters, and unmanned aerial vehicles, UAVs) for topography surveys. Table 1 reviews recent efforts that have attempted to use airborne platforms for landslide studies. Majority of the studies have exploited the optical data (red, green, blue bands) for landslide detection through image classification [[27], [28], [29], [30], [31], [32], [33], [34], [35]] or the aerial photogrammetry for landslide analysis [[36], [37], [38], [39]]. However, as the photogrammetry method measures the reflectance of sunlight from an object, it hardly captures the topographic information if the ground is obscured by trees and canopy in densely wooded or vegetated environments.

**Table 1:** Landslide-related studies using various sensors on airborne platforms.

Data type	Site characteristic	Method	Finding	Reference
• RGB images	• Forest	• Landslide detection	• The results has high accuracy and the effectiveness of predicting new potential landslides is demonstrated. • The results showed that the landslide inventory suggested a potentially important supplement to field mapping.	[27,28,[30], [31], [32], [33],^35^]
• RGB + Multi-spectral images	• Forest	• Landslide detection	• Continuous expansion of landslide scarps and movement of landslide head along the slope.	[29,34]
• RGB 3-D point cloud	• Hill • Cliff	• Landslide detection • Topographic change	• The slope portions being prone to failure and involved area and volume were calculated. • Understanding sediment provenance and transport. • Erosion and deposition of soils were detected.	[[36], [37], [38], [39]]
• LiDAR point cloud	• Forest	• Topographic change	• The sediment discharge was highly correlated with the flow length.	[40]
• LiDAR point cloud	• Forest	• Topographic data	• Landslide vulnerability and risk assessment.	[41]
• LiDAR point cloud	• Hill	• Topographic change	• Landslide area and volume were calculated from multiple LiDAR data. • Based on the data, they found the rupture of the retaining wall.	[42]
• LiDAR point cloud	• Cliff	• Topographic data	• Stability analysis of a blocky rock mass slope.	[43]
• Thermal infrared image	• Cliff	• Thermal change of permafrost slopes	• Thermal effect caused by engineering structures (i.e., highway, railway, electric tower, etc.) could increase the ground surface temperature and cause the underlying permafrost foundation to thaw.	[44]

On the other hand, to address the shortcomings of aerial photogrammetry, integration of LiDAR sensors with airborne platforms has been attempted in recent studies (Table 1). The LiDAR sensor emits an array of light sources at a range of the incident angle, such that the light transmits through the gaps between leaves and trees. Accordingly, LiDAR sensors can capture sufficient topographic information beneath forest canopies. While most studies have installed a LiDAR sensor on a fixed-wing aircraft, either unmanned or manned; their expensive cost and resource hamper repetitive monitoring of local specific regions using of those fixed-wing aircraft-LiDAR systems. Moreover, a high flight altitude of such aircraft systems, typically higher than 1 km, often results in poor resolution with a low density of the accumulated point clouds. By contrast, multi-rotor aerial vehicles (or multi-rotor drones; hereafter, UAVs) fly at relatively low flight altitudes, typically less than 500 m, which enables the acquisition of dense point clouds with high resolutions using the same LiDAR sensor. Developing and controlling a UAV-LiDAR system requires a certain level of knowledge and expertise on the robot operating system (ROS) and the position calibration, and hence, despite of numerous benefits, exploitation of the UAV-LiDAR system has been fairly limited in landslide research. Although several previous studies have applied the UAV-LiDAR system to non-vegetated sites, such as rocky mountains and mining sites, few studies have utilized this technology in highly vegetated or forested areas [23,[43], [45], [46], [47], [48]].

This study presents an advanced geomorphological survey system by installing a LiDAR sensor on an UAV (or drone), referred to as the *UAV-LiDAR system*. Additionally, an algorithm is suggested to delineate the topographic information from the acquired 3D LiDAR point clouds beneath canopy and vegetation in forest environments. Accuracy and performance of the developed UAV-LiDAR system was examined by test flight at a vegetated area and also compared with the UAV-photogrammetry method. Finally, two case studies that implemented the UAV-LiDAR system at fields are presented. The developed system was first implemented to a landslide site immediately after the landslide occurrence for forensic analysis of 2020 Gokseong landslide in South Korea. Second, the UAV-LiDAR surveys were conducted two times, before and after dredging, for sediment monitoring and maintenance of a debris-flow barrier installed in Pocheon, South Korea.

## 2 Methodology: Development of the integrated UAV-LiDAR system

### 2.1 Hardware setup

**Fig. 1:**
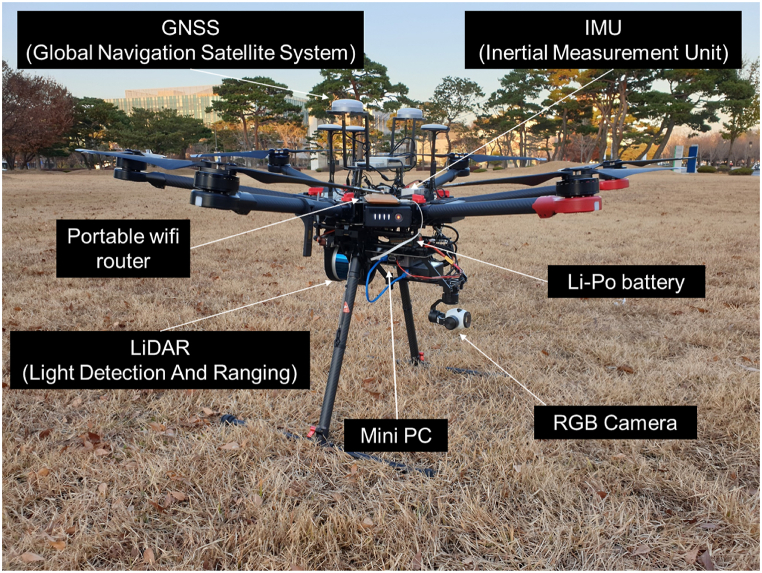
A photograph of the developed UAV-LiDAR system with all sensors installed.

**Fig. 2:**
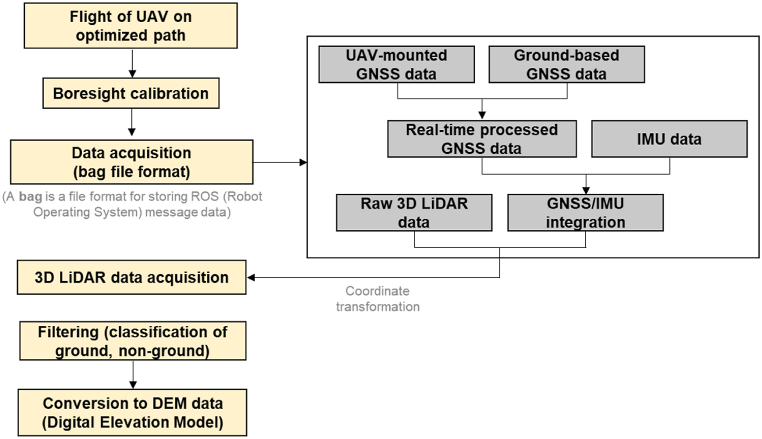
A flow chart for 3D LiDAR data acquisition and analysis.

Fig. 1 shows the developed UAV-LiDAR system with the sensors and peripheral electronics installed. The multi-rotor aerial vehicle (or UAV; DJI Matrice 600 Pro) houses a LiDAR sensor (Velodyne VLP-16) to collect high-resolution point clouds that reflect the positions of objects (*e.g.*, trees, ground, rocks, soil, artificial structures, etc.). Table 2 lists the specifications of the UAV-LiDAR system. The LiDAR sensor mounted by using a 3D printed fixture can scan the areas beneath the UAV along a perpendicular direction to the flight path. The LiDAR sensor generates laser pulses to measure the positions of objects based on a time-of-flight method; the LiDAR sensor used in this study can acquire 300,000 points/s for a single return mode with an accuracy of 3 cm and a maximum measurement distance of 100 m. The system includes a mini-PC (Intel NUC7i5BNK) as an onboard computer to control and manage the data acquisition during flight. In addition, there is a ground station composed of a DJI radio controller (RC) connected to a tablet PC, a laptop, and a real-time kinematic-global positioning system (RTK-GPS). The laptop communicates with the UAV through the RC to administrate the flight path and acquire the flight-related information (*e.g.,* flight path, altitude, and velocity), and it also communicates with the onboard computer to control the data acquisition process. Fig. 2 illustrates the flow chart for 3D LiDAR data collection and analysis.

**Table 2:** Specifications of the developed UAV-LiDAR system.

Type	Product	Company	Note
UAV	Matrice 600 pro	DJI	Maximum payload: 6 kg Maximum velocity: 65 km/h Maximum flight time: 38 min
			Horizontal accuracy: 1 cm Vertical accuracy: 2 cm
GNSS	RTK-GPS		
			Resolution: 12 MP
RGB Camera	Zenmuse Z3		
LiDAR	VLP-16 Puck	Velodyne	Measurement range: 100 m Wavelength: 905 nm Weight: 830 g Data acquisition: 300,000 points/sec
Mini PC	NUC7i5BNK	Intel	SSD (512 GB), memory (8 GB)
Li–Po battery	Graphene 2.0	Dinogy	14.8V, 2000mAh

The UAV (DJI Matrice 600 Pro) itself weighs 9.5 kg, and the maximum payload is 6 kg. The weight of the installed sensors, PC, and accessories is 2.4 kg; accordingly, the total weight of the developed UAV-LiDAR system is 12 kg. The UAV can fly at a speed up to 18 m/s, but this study mostly used the flight speed from 3 to 5 m/s for data quality. The hovering time of the UAV-LiDAR is estimated to range approximately 18–20 min with the payload of 2.4 kg; most of the field surveys in this study kept the flight time less than 15 min by taking into account the wind speed and the flight speed. The inertial measurement unit (IMU) and the GPS sensor installed in the UAV acquire the information on the x-, y- and z-positions, orientations (roll, pitch, yaw), flight altitude, and speed of the UAV. The precise positions and orientations of the UAV during flights enable accurate flight control and post-processing to extract the reliable positions of objects as point clouds. This study uses DJI RTK-GPS to detect the precise location of the UAV, which has the horizontal and vertical accuracies of 1 cm and 2 cm, respectively, and the maximum sampling frequency of 50 Hz (Table 2).

### 2.2 Position calibration of LiDAR point data

**Fig. 3:**
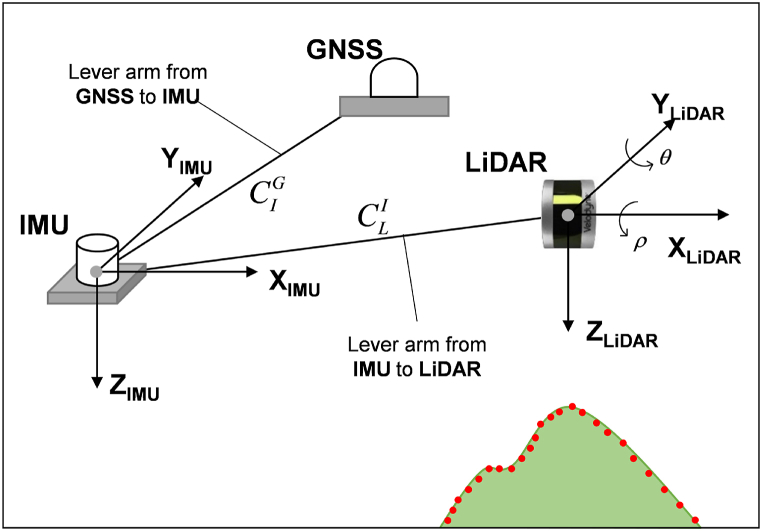
Translation of the LiDAR point clouds for the UAV orientation.

During flights, the LiDAR position also changes, following the position of the UAV. Accordingly, the raw point cloud acquired by the LiDAR need to be calibrated through translation into the global coordinate system (i.e., WGS 84), as shown in Fig. 3. The raw point cloud *P*_*S*_ is converted to the corrected point cloud with the actual position *P*_*t*_ with respect to the global coordinate system (WGS 84) by using the information on the orientation of the UAV acquired by IMU, as follows:

(1) $$P_{t}=R_{I}^{G}\left(R_{L}^{I}\cdot P_{S}+C_{L}^{I}\right)+C_{I}^{G},\text{and}$$

(2) $$P_{S}=\left[\begin{array}{ccc} \cos\;\rho & \sin\;\rho \;\sin\;\theta & \sin\;\rho \;\cos\;\theta \\ 0 & \cos\;\theta & -\sin\;\theta \\ -\sin\;\rho & \cos\;\rho \;\sin\;\theta & \cos\;\rho \;\sin\;\theta \end{array}\right]\left[\begin{array}{c} 0\\ 0\\ -r \end{array}\right]$$

where *P*_*t*_ is the corrected target point [*x y z*]^*T*^ in the mapping frame [North, East, and Altitude], *R*_*I*_^*G*^ is the rotation matrix from IMU to GNSS, *R*_*L*_^*I*^ is the rotation matrix from LiDAR to IMU, *C*_*L*_^*I*^ is the lever arm from LiDAR to IMU, *C*_*I*_^*G*^ is the lever arm from IMU to GNSS, *P*_*s*_ is the observed raw point [*x*_*o*_ *y*_*o*_ *z*_*o*_]^*T*^ by LiDAR, ρ is the roll angle, θ is the pitch angle, and *r* is the measured range.

### 2.3 Filtering of non-ground points

The acquired point clouds include topographic information (or ground points) and non-ground information, such as canopy, trees and vegetations. There are several approaches presented to capture the ground while filtering out the non-ground points, which include the slope-based filter [[49], [50], [51], [52]], morphological filter [[53], [54], [55], [56]], surface-based filter [57,58] and progressive morphological filter [54]. This study employs the cloth simulation filter (CSF; CloudCompare software, v.2.10.2) [59]. The CSF method requires relatively fewer parameters (three or four parameters), has good applicability to various land-cover types (*e.g.*, complex urban and steep mountainous areas), and directly works with raw LiDAR data [59]. A cloth is created by a grid model consisting of particles with mass and interconnections, referred to as a mass-spring model [60]. After turning the LiDAR data upside down, the virtual cloth falls on the LiDAR point data. External (i.e., gravity and collision forces) and internal forces (i.e., interconnections) are calculated for each node, segmenting the ground points from the non-ground points. Additional details of the CSF method can be found in Zhang et al. [59] and Zhang et al. [61]. The extracted ground point clouds are converted into a digital elevation model (DEM) using an interpolation method.

### 2.4 Benchmark accuracy test

**Fig. 4:**
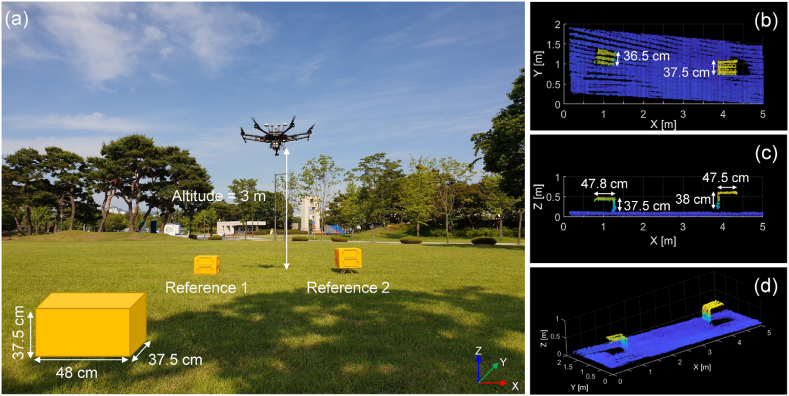
(a) Benchmark accuracy test setup with the reference boxes, (b) top view, (c) cross-section view, and (d) 3D view on the acquired 3D point clouds.

A benchmark test was carried out to assess the accuracy of the developed system, where the point clouds were obtained for the two boxes placed on ground, as shown in Fig. 4. The dimensions of the box were 375 mm wide, 375 mm long, and 480 mm high. One box was placed on the ground (Reference 1), and the other was placed at a height of 20 cm with a manual lab jack lift (Reference 2). The coordinates of the top center of the box were obtained using a GNSS device (Trimble R10) that had the horizontal and vertical accuracies of 8 mm and 15 mm, respectively. The UAV-LiDAR system acquired a 3D point cloud of the area while hovering at an altitude of 3 m from the ground (Fig. 4a).

Fig. 4b-to-4d show the point clouds obtained by the developed UAV-LiDAR system. The point cloud estimated the box width as 370–380 mm with an error of ±5 mm, which corresponds to the direction parallel to the spinning in the LiDAR sensor. In the perpendicular direction, the box length was measured as approximately 365–385 mm with an error of ±10 mm. For the vertical z-direction, the error ranged ±5 mm. Meanwhile, upon the boresight calibration, the position of the box according to the global coordinate system (WGS 84) obtained with the UAV-LiDAR system was remarkably consistent with that measured using the GNSS device.

## 3 Study sites and survey methods

In this study, the developed UAV-LiDAR system was employed in three sites: one test flight site and two field demonstration sites. A densely wooded hill was chosen for the test flight site; and one site where a landslide occurred and another site with a debris-flow barrier were chosen to prove the field applicability of the developed system.

### 3.1 Test flight site

**Fig. 5:**
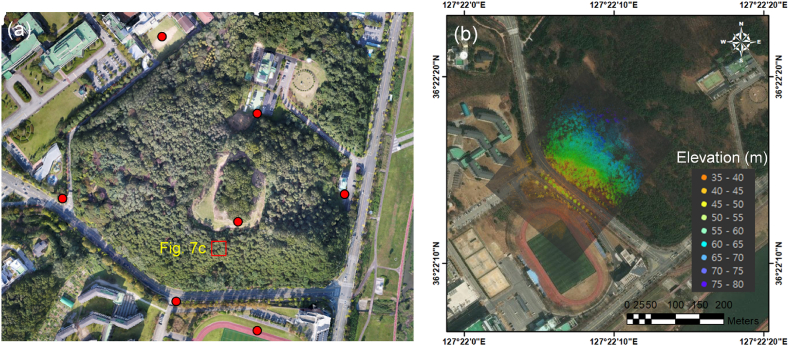
(a) Test flight site in Daejeon, Korea (36°22′16″N, 127°22′12″E) and (b) the acquired 3D LiDAR point clouds. Note that the red circles in Fig. 5a indicate the GCPs and the red rectangle indicates the specific test area of Fig. 7c.

The UAV-LiDAR system was first test-flown in a wooded hill located in Daejeon, South Korea (36°22′16″N, 127°22′12″E), as shown in Fig. 5. The maximum altitude of this hill was 100 m above the sea level. The UAV-LiDAR system was manually flown at an altitude of 110 m with a velocity of 3 m/s to acquire a 3D LiDAR point cloud of the surveyed area of approximately 200 m × 250 m (Fig. 5b). The acquired point clouds were calibrated for the UAV's position and orientation, and the CSF filter was used to filter the non-ground points [[59], [61]].

In addition, photogrammetry was performed to compare the results with the UAV-LiDAR system. Using a multi-rotor airborne platform, a total of 946 images of the test site were captured at an altitude of 150 m, with the front and side overlaps of 90% and 80%, respectively. Herein, the point clouds were extracted from the acquired images using the structure from motion algorithm (SfM; Agisoft Metashape software, v.1.5.5). The SfM algorithm uses the motion information of several two-dimensional images to trace the position and direction of the camera in reverse and then structures the relationship between the images and the camera [62,63]. The scale-invariant feature transform (SIFT) algorithm is used as the feature identification method to find the tie points (or features) while minimizing the noise effects by scale, illumination, or rotation for reverse tracing [64,65]. Here, the point clouds from the photogrammetry method were calibrated using the ground control points (GCP) shown in Fig. 5a [[36], [37], [38], [39]].

### 3.2 Field demonstration site 1: Gokseong landslide

**Fig. 6:**
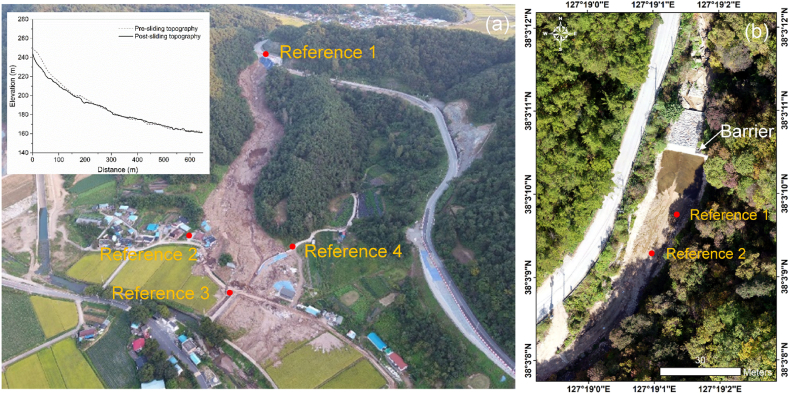
(a) An aerial photograph of the Gokseong landslide site and (b) RGB point clouds of the Pocheon barrier site before dredging. The red reference points denote the markers used to align the point clouds from the GNSS device and the UAV-LiDAR system.

At approximately 8:30 p.m. on August 7, 2020, a severe landslide event occurred in Osan town, Gokseong County, South Jeolla Province, South Korea, following three consecutive days of torrential rainfall, as shown in Fig. 6a (35°11′40″N, 127°8′10″E) [66]. The landslide involved a debris flow with a long runout distance and resulted in catastrophic damage to a nearby village located downstream. Choi et al. [66] reports more detailed information on this 2020 Gokseong landslide.

Five days later, a forensic field survey was conducted by deploying the UAV-LiDAR system at the Gokseong landslide site. The developed UAV-LiDAR system flied at the flight altitude of 300 m and the velocity of 3 m/s, respectively, and acquired 3D LiDAR point clouds of the landslide site.

### 3.3 Field demonstration site 2: Pocheon barrier maintenance

This study chose a site with a close-type barrier in Pocheon, South Korea as the second field demonstrate site, as shown in Fig. 6b (38°3′11″N, 127°19′1″W). The barrier was installed in 2000, and it was 28 m wide and 5.5 m high. Due to heavy rainfall in the recent months prior to the survey, there were large amounts of soils and rocks deposited behind the barrier, which almost exceeded the barrier capacity.

Following the dredging schedule, two surveys were conducted before and after the dredging with the UAV-LiDAR system and photogrammetry. It was noted that the dredging removed the deposited soils and rocks and instead there was a water pool in the reclaimed space behind the barrier. The LiDAR and RGB point clouds were acquired before and after the dredging. The developed UAV-LiDAR system operated at the flight altitude of 260 m and the velocity of 1 m/s. For the photogrammetry, the multi-rotor airborne platform was flown at altitudes of 527 m and 480 m before and after the dredging, respectively. The front and side overlaps and analysis method were the same with those at the test flight site. A total of 382 and 473 images were acquired before and after the dredging, respectively.

## 4 Results from test flight site

### 4.1 Acquisition of 3D point cloud of terrain surface

**Fig. 7:**
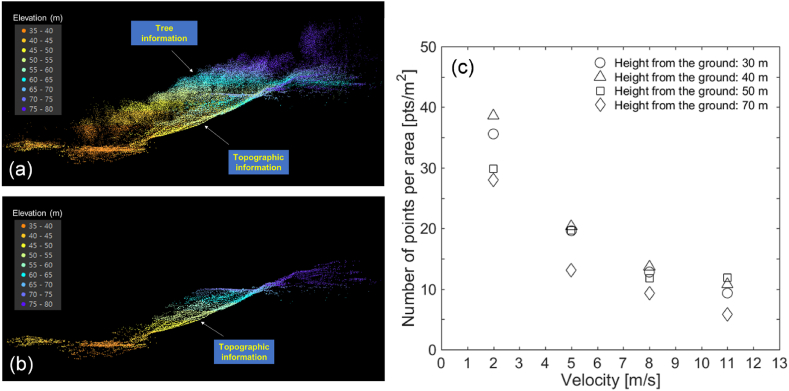
(a) Initial LiDAR point clouds, and (b) CSF-filtered LiDAR point clouds acquired from the wooded hill in Daejeon. (c) Changes in spatial density of point clouds with the altitude and velocity of the UAV.

The developed UAV-LiDAR system successfully acquired dense point clouds from the test site, as shown in Fig. 7a-to-7b. Not only the points reflected from trees (non-ground points), but the points reflected from the ground (ground points) were also acquired with a substantial density (Fig. 7a). The CSF filtering removed those non-ground points, and left only the ground points (Fig. 7b). Upon removal of the non-ground points by using the CSF filtering, there were sufficiently dense ground points, which enables to produce a high-resolution DEM of the terrain surface (or digital terrain model, DTM) with the cell size of less than 1 m for landslide analysis [[18], [19], [20], [21],^23^,[43], [45], [46], [47], [48]]. A movie on the LiDAR data acquisition during the flight can be found in Electronic Supplementary Materials (ESM1). Supplementary video related to this article can be found at https://doi.org/10.1016/j.heliyon.2023.e20225.

The following is/are the supplementary data related to this article:

Video S1 1 Video S1

### 4.2 Effect of flight velocity and height on point density

The effect of flight velocity and height on the point density was examined using the developed UAV-LiDAR system. In this study, the point density was defined as the number of ground points per unit area. The non-ground points were filtered out by using the CSF filter. The flights of the UAV-LiDAR system involved varying the flight velocity from 2 m/s to 11 m/s and changing the flight height from 30 m to 70 m. During each flight, the UAV-LiDAR system flied a straight path above the wooded hill at various velocities and altitudes. The result in Fig. 7c shows that the slower flight velocity produced the denser point cloud. At the height of 50 m, the point density was ∼12 pt/m^2^ at the flight velocity of 11 m/s, but it increased to ∼30 pt/m^2^ with the flight velocity of 2 m/s. The result shows that the lower flight height generated the denser point density, though its impact is less significant than the flight velocity. When the flight velocity was as slow as 2 m/s, the point density ranged from ∼30 pt/m^2^ at 70 m high to ∼35–40 pt/m^2^ at 30–40 m high. However, when the flight velocity was 11 m/s, the velocity-driven variation in the point density was the greater: 5 pt/m^2^ at 70 m high and 10–12 pt/m^2^ at 30–50 m high. Conclusively, it is preferable to fly the UAV at the slower velocity and the lower height, but note that this will reduce the coverage area and increase the flight operation time [[67], [68], [69], [70]].

The results provide the baseline data describing how the velocity and altitude of a UAV affect the spatial resolution. However, it should be noted that quantitative results are site-specific because the slope inclination and forest density vary from site to site. The presented methodology can be extended to determine the flight guide, including the flight velocity, altitude, and flight time for a UAV to acquire 3D LiDAR points with the required spatial resolution.

### 4.3 Comparison to UAV-photogrammetry

**Fig. 8:**
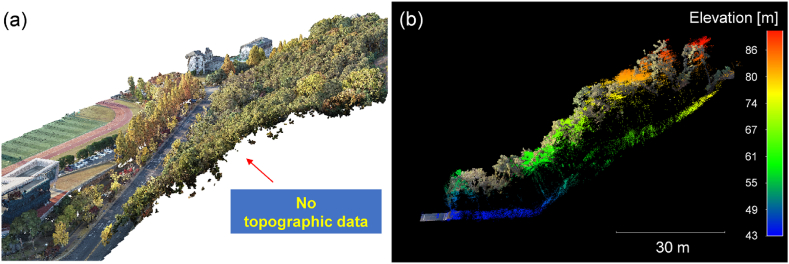
Photogrammetry result: (a) 3D RGB point clouds and (b) comparison with 3D LiDAR point clouds.

Fig. 8 compares the photogrammetry result with the UAV-LiDAR result. The trees were captured with dense points well. However, there were only minimal number of points capturing the ground (Fig. 8a) because the area was densely wooded and it limited sunlight transmission and reflection from the ground [[27], [28], [29], [30], [31], [32], [33], [34], [35]]. Fig. 8b highlights that the UAV-LiDAR system provided the richer point data from the ground, compared to the photogrammetry result [[43], [45], [46], [47], [48]]. Therefore, the UAV-LiDAR system proves its effectiveness in acquiring the terrain-relevant information, such as topographic information and relative ground displacement.

## 5 Field demonstrations

### 5.1 Site 1: Gokseong landslide site

**Fig. 9:**
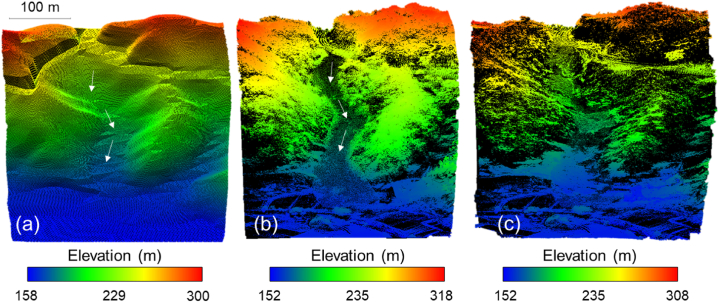
Digital elevation information: (a) before the event, (b) after the event without filtering and (c) after the event with filtering. Note that the white arrows indicate the landslide direction. The pre-event DEM was obtained from National Geographic Information Institute of Korea (NGII, 2020), and the post-event DEMs were obtained by using the UAV-LiDAR system.

Studying the characteristics of past landslides accurately aids in predicting and preparing for susceptible areas in the future [7,8,10,[12], [13], [14], [15], [16], [17],^71^]. Fig. 9 presents the digital elevation models (DEMs) before and after the landslide event in the Gokseong landslide site. The DEM before the event was acquired from National Geographic Information Institute of Korea (NGII, 2020, Fig. 9a). The DEMs after the event were obtained by using the developed UAV-LiDAR system (Fig. 9b -to-9c). Table 3 lists the coordinates of the reference locations for Site 1. Alignment of point clouds between GNSS and UAV-LiDAR system plays an important role in determining accuracy. The coordinate difference in Reference 3 of Site 1 is attributable to the absence of LiDAR points at the reference GNSS location. The vegetation-filtered 3D LiDAR point clouds after the event well capture the post-event terrain features. The 3D LiDAR point clouds estimate that the total length of the landslide path was ∼680 m and the initiation zone had a mean slope angle of 35°. The width of the landslide area ranged from 40 to 60 m at the initiation and transport zones, and the deposition fan had a maximum width of 140 m. Furthermore, the comparison between before-event and after-event topographic information enables characterization of the occurred landslide [[72], [73], [74], [75]]. The entrainment by the debris flow eroded the channel bottom by ∼2.5 m. Further details on the evidences and causes of the landslide and an illustration can be found in Choi et al. [66]. A movie on the LiDAR data acquisition during the flight can be found in Electronic Supplementary Materials (ESM2). Supplementary video related to this article can be found at https://doi.org/10.1016/j.heliyon.2023.e20225.

**Table 3:** Coordinates of the reference locations for Sites 1 and 2.

	Reference number	GNSS			UAV-LiDAR system		
		Latitude	Longitude	Altitude	Latitude	Longitude	Altitude
Site 1: Gokseong Landslide	1	35°11′40.98″N	127°8′10.71″E	250.87 m	35°11′41.97″N	127°8′10.71″E	250.90 m
	2	35°11′56.14″N	127°8′6.68″E	163.47 m	35°11′56.14″N	127°8′6.67″E	163.47 m
	3	35°11′58.23″N	127°8′3.17″E	162.41 m	35°11′58.23″N	127°8′3.12″E	163.26 m
	4	35°11′55.46″N	127°8′1.77″E	165.78 m	35°11′55.46″N	127°8′1.76″E	165.83 m
Site 2: Pochen barrier maintenance	1	38°3‘9.74″N	127°19’1.33″E	249.86 m	38°3‘9.74″N	127°19’1.33″E	249.82 m
	2	38°3‘9.29″N	127°19’0.92“E	251.24 m	38°3‘9.30″N	127°19’0.93“E	251.24 m

Note: The reference locations are denoted in Fig. 6.

The following is/are the supplementary data related to this article:

Video S2 2 Video S2

This field example demonstrates that the topographic information acquired immediately after the occurrence of a landslide are remarkably useful for post-event forensic analysis. Such topographic information can be used to determine not only the scale but also the cause of the landslide [[43], [45], [46], [47], [48],^72^,^73^,^75^,^76^]. However, as an access to the site right after the landslide event is typically restricted, it is fairly challenging to collect the necessary terrain-related information with limited access [72,[75], [76], [77], [78]]. The UAV-LiDAR system can overcome such restricted accessibility. In particular, the landslide initiation part is typically located on a steep slope and the vicinity of the initiated zone is prone to successive and subsequent failures. Meanwhile, the downstream parts are also challenging to access due to high water contents in soils. In addition, emergency recovery and restoration works may cause disturbance to the original terrain feature after the event (*e.g.*, landslide area, volume, rheological characteristics). Therefore, the UAV-LiDAR system can be effective in overcoming those limitations [72,[75], [76], [77], [78], [79], [80]]. As the GCP for the photogrammetry method is not required for the UAV-LiDAR system, the time and resources for survey can also be reduced [[43], [45], [46], [47], [48],^75^].

### 5.2 Site 2: Pocheon barrier site

**Fig. 10:**
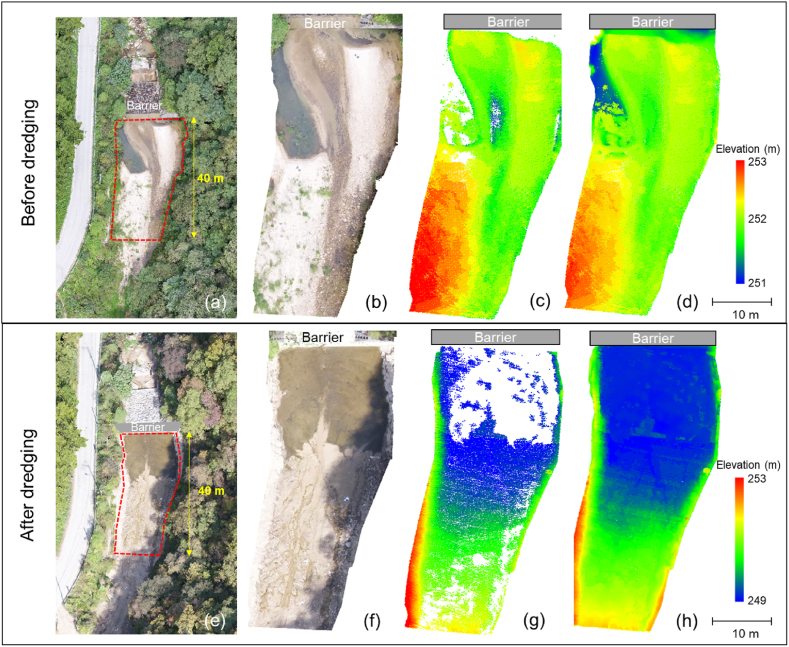
The survey results before the dredging (a-to-d) and after the dredging (e-to-f): (a,e) aerial photographs; (b,f) RGB point clouds; (c,g) LiDAR point clouds; (d,h) LiDAR point clouds filled with RGB point clouds.

Efficient management of debris-flow barriers is crucial to mitigate potential damage by landslides [9,[81], [82], [83], [84], [85]]. Fig. 10 shows the survey results on Site 2 before and after the dredging in the Pocheon barrier site. The survey covered the region 40 m upstream from the barrier, as shown in Fig. 10a-to-10e. In particular, the photogrammetry and LiDAR surveys provided the RGB point clouds (Fig. 10b-to-10f) and the LiDAR point clouds (Fig. 10c-to-10g) before and after the dredging. Table 3 shows the coordinates of the reference locations for Site 2. The CSF method filtered non-ground points from the LiDAR point clouds [59,61]. Note that there were no LiDAR points gathered from water and the soil with a high water content (Fig. 10c-to-10g). It is because water absorbs the laser emitted by the LiDAR sensor owing to its wavelength of 905 nm [86]. Instead, to fill these empty areas in the LiDAR point clouds, the RGB points obtained from the photogrammetry were utilized, as depicted in Fig. 10d-to-10h. Here, the RGB points at the water level were adopted to fill the empty spaces as the water depth was unknown.

**Fig. 11:**
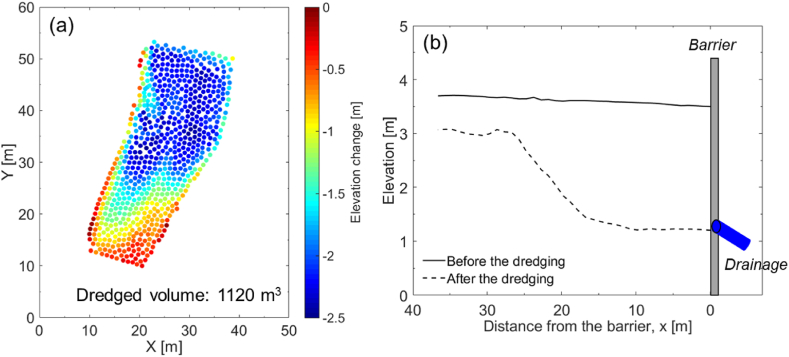
(a) Point clouds of the elevation changes, calculated using 3D LiDAR point clouds filled with RGB point clouds and (b) the pre-dredging and post-dredging ground elevation in this study.

Accordingly, the complete 3D point cloud datasets of the surveyed region were acquired before and after the dredging. These 3D point clouds enabled the estimation of elevation changes caused by dredging allowing the calculation of the total dredged volume. Fig. 11a shows the elevation change in the dredging area, estimated by using the M3C2 method [87,88]. The total dredged volume was estimated as 1120 m^3^, and the maximum elevation change was approximately −2.5 m close to the barrier (Fig. 11b). A movie on the LiDAR data acquisition during the flight can be found in Electronic Supplementary Materials (ESM3). Supplementary video related to this article can be found at https://doi.org/10.1016/j.heliyon.2023.e20225.

The following is/are the supplementary data related to this article:

Video S3 3 Video S3

This study used the photogrammetry as complementary data to the LiDAR point clouds because our LiDAR sensor (Velodyne VLP-16) was not effective to detect the ground covered with water or the soils with high water contents. This approach proved promising in monitoring sediment deposition near debris-flow barriers. Moreover, the benefit of using UAV-LiDAR systems becomes more prominent for the debris-flow barrier sites where the access is restricted or where it is densely wooded, such as in a mountainous region [72,[75], [76], [77], [78], [79], [80]]. Therefore, this can contribute to management and maintenance of the debris-flow barrier sites, where sediment transport actively takes place.

## 6 Conclusions

This study presents an integrated UAV-LiDAR system for geomorphological field survey by taking the advantages of LiDAR sensors and multi-rotor aerial vehicles. The test-flight over a vegetated area proves that the developed UAV-LiDAR system with a proper filtering method is capable of high-resolution topographic data acquisition in vegetated environments. For the given equipment capability, it is recommended to fly the UAV-LiDAR system at an altitude below 70 m from the ground and at a speed slower than 11 m/s for sub-0.5 m resolution. When the altitude is less than a certain distance from the ground, *e.g.,* within 70 m in this study, a lowered flight velocity is effective to increase the number of ground points captured and the resolution, while it needs to be balanced with the UAV battery management. The field demonstrations reveal that the survey with a UAV-LiDAR system is useful to acquire high-resolution topographic information at landslide sites that are not easily accessible. In addition, the LiDAR point clouds combined with photogrammetry point clouds can effectively capture grounds covered with water or soils with high water contents near a debris-flow barrier, which proves promising in monitoring sediment deposition in a mountainous region where the access is restricted or where it is densely wooded.

## Author contribution statement

Shin-Kyu Choi: Conceived and designed the experiments; Performed the experiments; Analyzed and interpreted the data; Wrote the paper. Ryan Angeles Ramirez: Performed the experiments; Wrote the paper. Tae-Hyuk Kwon: Conceived and designed the experiments; Analyzed and interpreted the data; Contributed reagents, materials, analysis tools or data; Wrote the paper.

## Data availability statement

Data will be made available on request.

## Declaration of competing interest

The authors declare that they have no known competing financial interests or personal relationships that could have appeared to influence the work reported in this paper.
